# Prevalence of tetracycline resistance genes among multi-drug resistant bacteria from selected water distribution systems in southwestern Nigeria

**DOI:** 10.1186/s12941-015-0093-1

**Published:** 2015-06-25

**Authors:** Ayodele. T. Adesoji, Adeniyi. A. Ogunjobi, Isaac. O. Olatoye, Douglas. R. Douglas

**Affiliations:** Department of Biological Sciences, Federal University Dutsin-Ma, Dutsin-Ma, Katsina State Nigeria; Department of Microbiology, University of Ibadan, Ibadan, Oyo State Nigeria; Department of Veterinary Public Health and Preventive Medicine, University of Ibadan, Ibadan, Oyo State Nigeria; Paul G. Allen School for Global Animal Health, Washington State University, Pullman, Washington State USA; Department of Veterinary Microbiology and Pathology, Washington State University, Pullman, USA

**Keywords:** Tetracycline resistance, Multidrug resistance, Water treatment, 16S rDNA library

## Abstract

**Background:**

Antibiotic resistance genes [ARGs] in aquatic systems have drawn increasing attention they could be transferred horizontally to pathogenic bacteria. Water treatment plants (WTPs) are intended to provide quality and widely available water to the local populace they serve. However, WTPs in developing countries may not be dependable for clean water and they could serve as points of dissemination for antibiotic resistant bacteria. Only a few studies have investigated the occurrence of ARGs among these bacteria including tetracycline resistance genes in water distribution systems in Nigeria.

**Methodology:**

Multi-drug resistant (MDR) bacteria, including resistance to tetracycline, were isolated from treated and untreated water distribution systems in southwest Nigeria. MDR bacteria were resistant to >3 classes of antibiotics based on break-point assays. Isolates were characterized using partial 16S rDNA sequencing and PCR assays for six tetracycline-resistance genes. Plasmid conjugation was evaluated using *E. coli* strain DH5α as the recipient strain.

**Results:**

Out of the 105 bacteria, 85 (81 %) and 20 (19 %) were Gram- negative or Gram- positive, respectively. Twenty-nine isolates carried at least one of the targeted tetracycline resistance genes including strains of *Aeromonas*, *Alcaligenes*, *Bacillus*, *Klebsiella*, *Leucobacter*, *Morganella*, *Proteus* and a sequence matching a previously uncultured bacteria. *Tet*(A) was the most prevalent (16/29) followed by *tet*(E) (4/29) and *tet30* (2/29). *Tet*(O) was not detected in any of the isolates. *Tet*(A) was mostly found with *Alcaligenes* strains (9/10) and a combination of more than one resistance gene was observed only amongst *Alcaligenes* strains [*tet*(A) + *tet30* (2/10), *tet*(A) + *tet*(E) (3/10), *tet*(E) + *tet*(M) (1/10), *tet*(E) + *tet30* (1/10)]. *Tet*(A) was transferred by conjugation for five *Alcaligenes* and two *E. coli* isolates.

**Conclusions:**

This study found a high prevalence of plasmid-encoded *tet*(A) among *Alcaligenes* isolates, raising the possibility that this strain could shuttle resistance plasmids to pathogenic bacteria.

## Introduction

Tetracycline antibiotics have been used to treat infectious diseases for more than half a century [1]; they have also been used nearly as long to promote growth in food animal production systems [2–4]. Growth-promoting properties of tetracyclines were first described in 1949 for chickens fed chlortetracycline supplemented feed [5]. Subsequently, they were widely applied in animal husbandry thanks to improving the growth rate to feed intake ratio [6–8].

Tetracycline inhibits bacterial growth by interfering with protein synthesis when the antibiotic binds to the 30S ribosomal subunit thereby preventing aminoacyl-t-RNA binding to the ribosomal A site and preventing synthesis of polypeptides [1]. Resistance to tetracycline is usually conferred through acquisition of resistance genes associated with mobile genetic elements [9]. These genes could be disseminated by interspecies transfer mediated by plasmids, transposons, and bacteriophage [10, 11]. Once resistance genes are introduced into the environment, they are also exposed to selective pressure, such as antibiotics produced by indigenous antibiotic producers in soil. However, selection can occur in the environment without antibiotic selective pressure [12]. For example, Gilliver *et al.* [13] reported that antibiotic-resistant bacteria were found in wild rodents that had never been exposed to antibiotics. Therefore, antibiotic resistance genes might be distributed and preserved in the broader environment with or without antibiotic selective pressure. Four resistance mechanisms have been described, including protection of the ribosome by a large cytoplasmic protein, energy-dependent efflux, enzymatic inactivation and target modification [14, 15].

Several investigators have reported findings for antibiotic resistant bacteria found in finished drinking water based on culture-dependent methods [16], indicator organisms [17] and qualitative and quantitative molecular techniques [18, 19, 16]. Studies have demonstrated that the susceptibility of antibiotic resistant bacteria to a disinfectant and the susceptibility of antibiotic-susceptible bacteria to a disinfectant can be genetically linked [20, 21] and thus co-selection can occur with disinfectant exposure. Armstrong et al. [22, 23] suggested that stress-tolerant bacteria selected by chlorination might be more antibiotic resistant, and one study found that suboptimal chlorine treatment of drinking water selected for multidrug-resistant *Pseudomonas aeruginosa* [24]. Xi *et al.* [16] reported an increased prevalence of antibiotic resistance genes and specialized groups of antibiotic resistant bacteria in tap water compared to source water and they, suggested that water treatment could differentially favor the antibiotic resistant bacteria and/or induce transfer of antibiotic resistance genes among subsets of the bacterial population. Figueira *et al.* [25] studied different populations of waste water *E. coli* and concluded that variations on the prevalence of quinolone resistance were correlated with the dynamics of some population sub-sets. Vaz-Moreira *et al.* [26] characterized patterns of antimicrobial resistance for sphingomonads isolated from tap water and cup fillers of dental chairs. They also concluded that antibiotic resistance patterns are often species- rather than site-related. There is a paucity of information on the prevalence of tetracycline resistance genes among bacteria isolates from treated and untreated water in Nigeria. The goal of this study was to estimate the prevalence of select tetracycline resistance genes among bacteria from isolated water distribution systems in south-west Nigeria.

## Materials and methods

### Site description

Samples were collected from water treatment plants (WTPs) that employ conventional methodologies for water purification including filtration, flocculation, sedimentation and disinfection. The Ife dam is the smallest (0.95 km^2^) and is located at Obafemi Awolowo University Ile-Ife, Osun State. The Ede Erinle dam is located in Ede, Osun State and extends approximately 14.0 km^2^ at the normal water level. The Eleyele dam (1.5 km^2^) services the Ibadan metropolis in Oyo State Nigeria. The Asejire dam (7.5 km^2^) is located in Asejire, approximately 30 km east of Ibadan. The Owena-Ondo dam (7.8 km^2^) is located near Akure town, Ondo State. The Owena-Ijesha dam (1.7 km^2^) is located near Ilesha, in Osun State. More details about each of these sample sites can be found elsewhere [27–29].

#### Sample collection, bacteria isolation and characterization

Water samples were collected into sterile bottles from raw, treated and municipal taps at each WTPs. Samples were collected four times during a seven month period (December, 2010 to July, 2011). Samples were serially diluted and plated on Nutrient agar, Eosin Methylene blue agar (EMB) and Deoxycholate agar (DCA). Afterwards, bacteria were picked with the goal of maximizing the diversity of colony morphology represented from each sample. Picked colonies were re-streaked on Nutrient agar to obtain pure cultures. These were subsequently transferred to Nutrient agar slants and also stored in phosphate buffer glycerol at −80 °C.

#### Identification of bacteria using 16S rDNA sequencing

Total genomic DNA was prepared from isolates after overnight culture in Luria Betani (LB) agar. A sample of culture was selected using a sterile plastic loop and re-suspended in 200 μl of 5 % Chelex 100 (BioRad) in a microcentifuge tube. The suspensions were boiled for 10 min, followed by centrifugation for 1 min (14, 000 ×*g*). A fragment of the 16S rDNA sequence was amplified using primer 16 s-8 F (AGAGTTTGATCMTGGCTCAG) and 16 s-517R (ATTACCGCGGCTGCTGG) [30, 31]. Extracted DNA supernatant (5 μl) was used as template with 2 mM MgCl_2_, 0.8 mM dNTPs, 0.2 μM of each primer and 1X PCR buffer. Reaction conditions included 1 min denaturation (95 °C) followed by 30 cycles of 96 °C for 30 s, 60 °C for 30 s and 72 °C for 30 s and a final extension of 72 °C for 10 min. PCR products were separated and visualized by gel electrophoresis (1 %) to confirm amplification. PCR products were sequenced using Big Dye Terminator chemistry (Eurofins MWG, USA) and manual base calls and sequence trimming was completed using Sequencher 5.0 (Gene Codes Corporation, Ann Arbor, MI) BLASTn [32] was used to identify the best matches from Genbank based on percent sequence identity.

#### Antibiotic susceptibility testing

The antibiotic resistance profile of the bacteria was determined using breakpoint assays on LB Agar plates. Agar was autoclaved, cooled to 55 °C and antibiotics were added to specific breakpoint concentration (Table 1) before agar was poured into petri dishes (150 × 15 mm). Bacteria were retrieved from freezer stocks and transferred from 96-well plates into another 96-well plate with sterile LB broth using a pin replicator and incubated overnight at 37 °C. Cultured isolates were then re-transferred onto LB plates with the antibiotics and incubated over night at 37 °C. Isolates were scored as “1” for growth (resistant) or “0” for no growth (susceptible) for each antibiotic plate. A no-antibiotic plate was used to confirm successful transfer of culture to agar plates using the 96-well replicator. Isolates of bacteria that were resistant to >3 classes of antibiotics were considered MDR. Proportions of resistant bacteria were calculated and 95 % confidence intervals were estimated based on the formula 1.96*sqrt[(p*(1-p))/n] where *p* = proportion resistant and *n* = number of isolates resistant to a given antibiotic.

**Table 1:** Antibiotic concentrations tested against Gram-positive and Gram-negative bacteria

Antibiotics for gram negative with concentration (μg/ml)			Antibiotics for gram positive with concentration (μg/ml)		
Code	Name	Concentration	Code	Name	Concentration
FF	Florfenicol	16	SU	Sulfamethoxazole	512
T	Tetracycline	16	AM	Ampicillin	0.5
S	Streptomycin	16	T	Tetracycline	16
G	Gentamycin	16	SXT	Sulfamethoxazole/Trimethoprim	76/4
K	Kanamycin	64	G	Gentamycin	16
C	Chloramphenicol	32	E	Erythromycin	8
N	Nalidixic Acid	30	RIF	Rifampin	4
AMC	Amoxillin/Clavulanic Acid	32/16	LIN	Lincomycin	4
CEF	Ceftiofur	12	CIP	Ciprofloxacin	4
SU	Sulfamethoxazole	512			
SXT	Sulfamethoxazole/Trimethoprim	76/4			

#### Genotyping of tetracycline resistance genes

The diversity of tetracycline resistant genes encoded in the genome of 105 tetracycline and multidrug resistant isolates was assessed by testing for the presence of tetracycline resistance genes that encode resistant by efflux pump mechanism [*tet*(A), *tet*(B), *tet*(E), *tet*30] and genes that encode ribosomal protection proteins [*tet*(M) and *tet*(O)] (Table 2). PCR amplification reactions included 5 μl of the Chelex extracted DNA as template. The PCR reaction mixture followed the same protocol as described for 16S rDNA PCR with the exception of different primer annealing temperature (Table 2).

**Table 2:** Primers used in this study for amplification of tetracycline-resistance genes (Call et al., 2003)

Primer pair	Target	Sequence (5′-3′)	Annealing temperature (°C)	Amplicon size (bp)	Reference
tet(A)- F	*tet*(A)	TTGGCATTCTGCATTCACTC	60	494	Call *et al.,* 2003
tet(A)- R		GTATAGCTTGCCGGAAGTCG	60	494	,,
tet(B)- F	*tet*(B)	CAGTGCTGTTGTTGTCATTAA	60	571	,,
tet(B)- R		GCTTGGAATACTGAGTGTAA	60	571	
tet(E)- F	*tet*(E)	TATTAACGGGCTGGCATTTC	55	544	,,
tet(E)- R		AGCTGTCAGGTGGGTCAAAC	55	544	,,
tet(M)- F	*tet*(M)	ACACGCCAGGACATATGGAT	55	536	,,
tet(M)- R		ATTTCCGCAAAGTTCAGACG	55	536	,,
tet(30)- F	*tet*(30)	CCGTCATGCAATTTGTGTTC	55	550	,,
tet(30)- R		TAGAGCACCCAGATCGTTCC	55	550	,,
Tet(O)-F	*tet*(O)	GCGGTAATTATGGGAAACGA	55	550	,,
Tet(O)-R		TTTCCCGCTGTTCAGATTTC			

### Conjugation experiments

A nalidixic acid resistant strain of *E. coli* (DH5α) was used as a recipient for conjugation experiments. A subset of isolates (*n* = 19) was selected as donor strains because they harboured a diversity of tetracycline resistance genes (*tet* (A)*, tet* (B) and *tet* 30) while being sensitive to nalidixic acid. Overnight cultures were prepared in LB broth. Nitrocellulose membrane papers were cut (2 × 1 cm) and were sterilized by using a dry cycle autoclave at 121 °C for 30 min. The sterile filters were placed aseptically on the surface of solidified sterile LB agar. Overnight broth cultures (10 μl) of both the donor and recipient bacteria were spotted together onto a filter paper and were incubated overnight at 37 °C. Thereafter, 500 μl of sterile phosphate buffer saline was used to wash the bacteria from the nitrocellulose paper into a sterile petri dish. A micropipette was used to transfer the fluid on to the surface of LB agar plate containing 20 μl/ml of nalidixic acid and 16 μl/ml of tetracycline followed by overnight incubation. When bacteria grew under this condition they were considered successful conjugants. DNA was extracted from representative isolates using Chelex method (described above) and used in the PCR amplification of the transferred genes for genotypic confirmation.

## Results and discussion

**Fig 1:**
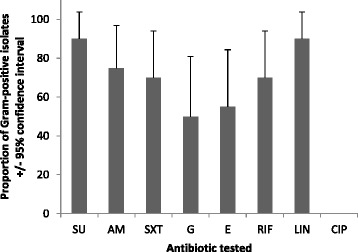
Prevalence of antibiotic resistant Gram-positive bacteria from all sampled water (+95 % confidence intervals). Codes: Ampicillin (AM); Gentamycin (GEN); Sulfamethoxazole (SU); Sulfamethoxazole/ Trimethoprim (SXT); Erythromycin (E); Riframprim (RIF); Lincomycin (LIN); Ciprofloxacin (CIP)

**Fig 2:**
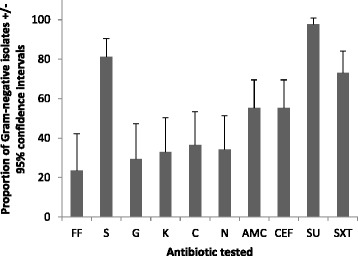
Prevalence of antibiotic resistant Gram-negative bacteria from all sampled water (+95 % confidence intervals). Codes: Ceftiofur (CEF); Chloramphenicol (C); Florfenicol (FF); Kanamycin (K), Streptomycin (S) and Gentamycin (GEN); Nalidixic Acid (N); Sulfamethoxazole (SU); Sulfamethoxazole/ Trimethoprim (SXT); Amoxicillin/Clavulanic Acid (AMC)

Details on the physicochemical, microbial quality and antibiogram based on sample location of each of these water distribution systems have been reported elsewhere [27–29]. Herein we report findings for a total of 105 multi-drug resistant (MDR) bacteria that included strains with resistance to tetracycline that were selected for genotyping. Eighty-two (80.95 %) and 23 (19.04 %) of isolates were Gram-negative and Gram-positive, respectively. We could discern no outstanding differences between sample sites that might contribute to the variance within and between these locations. For Gram-positive bacteria resistance was detected for all antibiotics tested except ciprofloxacin (Fig. 1) whereas resistant isolates were detected for all antibiotics tested for Gram-negative bacteria (Fig. 2). The absence of ciprofloxacin resistance for the Gram-positive isolates might reflect a relatively low-level of use in Nigeria [33] or we may have adopted a breakpoint concentration that was too high to detect variation in resistance levels.

Twenty nine isolates from all sample locations possessed tetracycline-resistance genes with *tet*(A) being the most prevalent (Table 3). The two bacteria carrying *tet*30 were isolated from the Ede finished water and Owena-Idanre municipal tap water. 16SrDNA sequencing indicated that these two bacteria were members of the *Alcaligenes* genera (Table 4). This may be the first report of *tet*30 in this genera indicating that this gene can be found among a wide diversity of bacteria [9].

**Table 3:** Summary of total numbers of isolates collected per sample during the study and number positive for each tet genotype

Sample site	*No* + ve for at least one *tet* gene	*tet*(A)	*tet*(B)	*tet*(E)	*tet*(M)	*tet30*	Total
Ife, Dam 1							
Raw water	2	1	0	1	0	0	5
Finished water	3	1	0	1	1	0	7
Tap water	0	0	0	0	0	0	2
Tap water	0	0	0	0	0	0	0
Ede, Dam 2							
Raw water	1	0	1	0	0	0	5
Finished water	2	2	0	0	0	1	3
Tap water	0	0	0	0	0	0	0
Tap water	0	0	0	0	0	0	1
Asejire Dam 3							
Raw water	3	1	2	ND	ND	ND	7
Finished water	0	0	0	0	0	0	6
Tap water	1	0	1	0	0	0	3
Tap water	0	0	0	0	0	0	1
Eleyele Dam 4							
Raw water	1	0	0	0	1	0	11
Finished water	0	0	0	0	0	0	0
Tap water	1	0	0	1	0	0	1
Tap water	0	0	0	0	0	0	0
Owena-Ondo Dam 5							
Raw water	0	0	0	0	0	0	0
Finished water	3	3	0	0	0	0	5
Tap water	1	1	0	0	0	0	6
Tap water	3	3	0	0	0	0	7
Tap water	1	0	0	0	1	0	8
Owena-Ijesha Dam 6							
Raw water	3	1	0	2	2	0	17
Finished water	0	0	0	0	0	0	1
Tap water	2	1	0	1	0	0	5
Tap water	2	2	0	2	0	1	4
Total	29	16	4	8	5	2	105

**Table 4:** Summary of prevalence and total number of tetracycline-resistant bacteria species and genotypes

Bacteria	No of MDR isolates genotyped	No. (%) tetracycline resistant	Source of Isolate	*tet* genotype	No Positive for *tet* gene
*Aeromonas spp*	5	1 (3.44)	IRW	*tet*(E)	1
*Alcaligenes spp*	20	10 (34.48)	IFW, EDFW, OWODFW,OWODM2, OWIRW, OWIM1, OWIM2	*tet*(A)	9
				*tet*(E)	4
				*tet*(M)	1
				*tet30*	2
				*tet*(A) *+ tet30*	2
				*tet*(A) *+ tet*(E)	3
				*tet*(A) *+ tet*(M)	1
				*tet*(E) *+ tet*(M)	1
				*tet*(E) *+ tet30*	1
				*tet*(A) *+ tet*(E) *+ tet30*	1
				*tet*(A) *+ tet*(E) *+ tet*(M)	1
				*tet*(A) *+ tet*(E) *+ tet*(M)	1
*Bacillus spp*	45	2 (6.89)	IFW, EDRW	*tet*(B)	1
				*tet*(E)	1
*Klebsiella spp*	14	1 (3.44)	OWIRW	*tet*(E)	1
*Leucobacter spp*	2	1(3.44)	ARW	*tet*(B)	1
*Morganella spp*	7	6 (20.69)	EDFW, ERW, OWODFW, OWODM1, OWODM3, OWIRW	*tet*(A) + *tet*(M)	3
					3
*Proteus spp*	22	2 (6.90)	ARW, EFW	*tet*(A)	1
				*tet*(B)	1
Uncultured bacteria clone	7	3 (10.34)	ARW, AM1	*tet*(B) + *tet*(M)	2
					1

Codes: IRW = Ife raw water, IFFW = Ife treated water, IFM1 and IFM2 = Ife municipal tap 1 and 2, EDRW = Ede raw water, EDFW = Ede treated water, EDM1 and EDM2 = Ede municipal tap 1 and 2, ARW = Asejire raw water, AFW = Asejire treated water, AM1 and AM2 = Asejire municipal tap 1 and 2, ERW = Eleyele raw water, EFW = Eleyele treated water, EM1 and EM2 = Eleyele municipal 1 and 2, OWODRW = Owena Ondo raw water, OWODFW = Owena Ondo treated water, OWODM1 and OWODM2 = Owena-ondo municipal tap 1 and 2, OWIRW = Owena-Idanre raw water, OWIFW = Owena-Idanre treated water, OWIM1 and OWIM2 = Owena-Idanre municipal tap 1 and 2 Note: Bacteria was identified to the genus level by 16S rDNA partial Sequence

Two Gram-positive (*Bacillus* sp and *Leucobacter* sp) and three Gram-negative bacteria (1 *Proteus* and 2 unidentified) harbored *tet*(B) (Table 4). This gene has been reported previously in *Bacillus* and *Proteus* [34] but this may be the first report for detection of *tet*(B) for the genus *Leucobacter*. Sequence analysis of the *tet* (B) gene showed that it was 99 % similar to *tet*(B) gene found in a *Pseudomonas* strain (accession no: AB089594.1). We surmise, however, presence of this gene in an unidentified environmental isolate implies that unidentified bacteria from the environment could be a reservoir and source of transfer to other bacteria of antibiotic resistance gene. This is similar to the report of Riesenfeld *et al.* [35] who also detected antibiotic resistance genes attributable to uncultured or unidentified soil bacteria.

The *tet*(E) gene was detected amongst a diverse group of genera (*Aeromonas*, *Bacillus*, *Klebsiella*, *Leucobacter* and *Alcaligenes*; Table 4). Jacobs and Chenia [36] reported that *tet*(E) is frequently detected among the *Aeromonas* in South Africa. Chopra and Roberts [11] reported that *tet*(E) has been found in combination with *tet*(B), *tet*(I), *tet*(C) *tet*(D), *tet*31 in the same bacteria including additional genera (*Edwardsiella*, *Providencia*, *Proteus*, *Citrobacter*, *Shigella)*. We only found *tet*(E) in conjunction with *tet*(M)*, tet*(A) and *tet*30 in *Alcaligenes* isolates (Table 4). Other combinations of tetracycline-resistance genes were observed in the current study for the genus *Alcaligenes* (Table 4) and *Alcaligenes* was the only genus with >1 tetracycline gene present.

The higher prevalence of *tet*(A) (Table 4) compared to other tetracycline resistance genes is consistent with other reports [37, 38, 34]. Multiple reports indicate that the presence of these genes among different bacteria including *E.coli* [39] and *Aeromonas* [40]. Agerso and Sandvang [41] reported *tet*(A) for the first time in three isolates of *Alcaligenes*. Bacteria of the *Alcaligene* genus have been isolated from intestine of humans and from various hospital or environmental water sources [42]. These strains can produce opportunistic infection, particularly for severely immune-compromised patients including those with neutropenia and malignant tumor or cardiovascular disease [43, 44].

Ribosomal protection protein genes were detected less frequently in this study; we did not detect *tet*(O) while five bacteria were PCR-positive for *tet*(M). Another study showed that *tet*(M) was widely distributed in coastal aquaculture areas and sediments in Mekong River, Vietnam [45]. *Tet*(M) has been found in a diversity of bacteria including *Eikenella*, *Kingella* and *Neisseria* [11]. We found *tet*(M) in *Alcaligenes*, *Morganella* and an unidentified bacterium. The distribution of *tet*(M) no well described for bacteria that have less clinical significance.

There are numerous differences between each of the sample sites that preclude drawing associations between the presence of specific resistance genes relative to different WTP practices. It was notable however, that amongst the six water distribution systems, bacteria from Dam 5 (Owena-Ondo) carried the highest proportion of *tet*(A) (7/16) (Table 3). We also observed that out of 8 bacteria tested positive for *tet*(E), the highest (5/8) prevalence was among bacteria from Dam 6 (Owena-Ijesha), *tet*(B) was found among three isolates from the raw water of Asejire and Ede water treatment plants while none of the bacteria isolated from the treated water showed the presence of this gene. Experimental manipulation and controls would be needed to determine if different WTP practices contribute to differences in the distribution of tetracycline resistance genes.

We also tested other tetracycline-resistant MDR bacteria, including strains of *Acinetobacter*, *Aquitalae*, *Bordetella*, *Brevundimonas*, *Chromobacterium*, *Citrobacter*, *Camamonas*, *Enterobacter*, *Lysinibacillus*, *Myroides*, *Pantoae*, *Providencia*, *Pseudochrobactrum*, *Psychrobacter*, *Serratia*, *Sphingobacterium*, *Staphylococcus*, *Stenotrophomonas*, *Ralstonia* and *Trabulsiella* but we did not detect any of the tetracycline-resistance genes for which we tested (data not shown). It is likely that these isolates harbor a diversity of other tetracycline-resistance gene; earlier studies identified 23 genes encoding efflux pumps and 11 genes encoding ribosomal protection proteins, not including mosaic tetracycline resistance genes [46], since the first report of transferable tetracycline resistance in 1960 [9]. It is likely that other novel tetracycline-resistance genes can be found in these environments.

Conjugation experiments showed that five of 17 *tet*(A) positive bacteria successfully transferred the gene to the recipient host using the conditions employed in this study (three *Alcaligenes* and two *E. coli*; Table 5). Agerso and Sandvang. [41] demonstrated the transfer of *tet* (A) from *Alcaligenes* to *E.coli* from their studies, results consistent with the *tet*(A) gene being present on a broad-host plasmid(s) that this is capable of moving between genera. Agerso *et al.* [47] reported that *tet* (*A*) can be located on conjugative plasmids from different incompatibility groups. The one *tet*30-positive strain of *Alcaligenes* was able to transfer it to the recipient strain. Among the four bacteria carrying *tet*(*B*), only the *Proteus* could transfer this gene.

**Table 5:** Bacteria with transferrable tetracycline resistance genes and resistance phenotypes

Strain ID^a^	Bacteria/ascension no^b^	Source^c^	Tetracycline resistance gene transferred	Resistance phenotypes^d^
197	*Alcaligenes faecalis* JN162124.1	OWODFW	*Tet*(A)	T, S, K, CEF, AM, SXT, SU
198	*Alcaligenes sp*. JF707602.1	OWODFW	*Tet*(A)	T, S, K, AM, SXT, SU
173	*Alcaligenes faecalis* JN162124.1	OWIRW	*Tet*(A)	T, S, CEF, SXT, AMC, SU
210A	*Escherichia coli* CP003034.1	OWODM2	*Tet*(A)	T, AM, AMC, SU
210B	*Escherichia coli* CP003034.1	OWODM2	*Tet*(A)	T, AM, SXT, AMC, SU
46	*Proteus vulgaris* JN630888.1	ERW	*Tet*(B)	FF, T, S, G, K, C, AM, SXT, N, AMC, SU
28A	*Alcaligenes faecalis* HM145896.1	EDFW	*Tet30*	T, S, G, K, N, CEF, AM, SXT, SU

^a^This is our study specific ID designation

^b^Bacteria were identified by 16S rDNA partial sequencing while accenssion number is the Genbank number for the closest match

^c^For source of bacteria and code go to Table 3 and footnote of Table 4 respectively

^d^Codes: Ampicillin (AM); Ceftiofur (CEF); Chloramphenicol (C) and Florfenicol (FF); Kanamycin (K), Streptomycin (S) and Gentamycin (GEN); Tetracycline (T); Nalidixic Acid (N); Sulfamethoxazole (SU); Sulfamethoxazole/ Trimethoprim (SXT); Amoxicillin/Clavulanic Acid (AMC); Erythromycin (E); Riframprim (RIF); Lincomycin (LIN); Ciprofloxacin (CIP)

In summary, we detected transferrable tetracycline- resistance genes among a diversity collection of bacteria, *tet*(A) was the most common gene detected especially from isolates from the *Alcaligenes* genus, and horizontal transfer was verified by conjugation experiments. Given these findings it is possible that transfer of these genes occurs regularly in water distribution system. Moreover, Gao *et al.* [48] noted that antibiotic-resistant bacteria could be introduced into the food chain via aquaculture products and, presumably via washing of food products.
