# Erratum to: Prevalence of tetracycline resistance genes among multi-drug resistant bacteria from selected water distribution systems in southwestern Nigeria

**DOI:** 10.1186/s12941-015-0099-8

**Published:** 2015-09-11

**Authors:** Ayodele T. Adesoji, Adeniyi A. Ogunjobi, Isaac O. Olatoye, Douglas R. Call

**Affiliations:** Department of Biological Sciences, Federal University Dutsin-Ma, Dutsin-Ma, Katsina State Nigeria; Department of Microbiology, University of Ibadan, Ibadan, Oyo State Nigeria; Department of Veterinary Public Health and Preventive Medicine, University of Ibadan, Ibadan, Oyo State Nigeria; Paul G. Allen School for Global Animal Health, Washington State University, Pullman, WA USA; Department of Veterinary Microbiology and Pathology, Washington State University, Pullman, USA

## Erratum to: Annals of Clinical Microbiology and Antimicrobials (2015) 14:35 DOI 10.1186/s12941-015-0093-1

After publication of the original article the authors realised that Douglas. R. Call’s name was displayed incorrectly. The correct spelling of his name is included in this erratum; the correct spelling has also been updated in the original article. The authors realised that in Line 347—“AAT” should have been “ATA”, and “OAA” should have been “AAO”. In Line 348—“OIO” should have been “IOO”, “CRD” should have been “DRC” and “AAT” should have been “ATA”. In Line 349—“OIO” should have been “IOO” and “CRD” should have been “DRC”. In Line 325—“47” should have been “41”. In Line 329—“48” should have been “47”. In Line 340—“49” should have been “48”. In Line 503 reference ‘47’ should be removed. In Line 506—‘48’ should have been ‘47’ and in Line 510—reference ‘49’ should have been ‘48’.

